# Formulation Characteristics of Solid-Dispersible Self-Emulsifying Drug Delivery Systems for Dual Drug Delivery

**DOI:** 10.3390/pharmaceutics18060637

**Published:** 2026-05-22

**Authors:** Shailvi Soni, Terrick Andey

**Affiliations:** Department of Pharmaceutical Sciences, School of Pharmacy, Massachusetts College of Pharmacy and Health Sciences (MCPHS) University, 19 Foster St., Worcester, MA 01608, USA

**Keywords:** self-emulsifying drug delivery, freeze-dried double emulsion, lipid-based formulation

## Abstract

**Background**: Oral delivery of chemotherapeutic agents remains challenging due to gastrointestinal degradation, poor intestinal permeability, and extensive first-pass metabolism, which collectively limit bioavailability. Lipid-based drug delivery systems offer a promising strategy to overcome these barriers. This study aimed to develop a freeze-dried, solid-dispersible self-emulsifying drug delivery system (SEDDS) using a water-in-oil-in-water (w/o/w) double emulsion approach for the co-encapsulation of hydrophilic (doxorubicin) and lipophilic (ellipticine) agents to enhance oral delivery. **Methods**: Double-emulsion SEDDS were prepared via a two-stage emulsification process to enable compartmentalized drug loading within aqueous and oil phases. The formulations were freeze-dried to improve stability and storage. Physicochemical properties were characterized using dynamic light scattering for droplet size and polydispersity index (PDI), zeta potential analysis for colloidal stability, and differential scanning calorimetry for thermal behavior. Drug encapsulation efficiency was determined, and cellular uptake was evaluated in breast cancer cells using fluorescence microscopy. **Results**: Optimized SEDDS exhibited droplet sizes of 90–347 nm with low PDI values (0.005–0.336), indicating uniform and stable dispersions. Zeta potential values (−10.64 to 2.38 mV) supported colloidal stability, while freeze-dried formulations retained dispersion characteristics upon reconstitution over extended storage. Both drugs demonstrated high encapsulation efficiency (>97%), and thermal analysis confirmed the formation of stable amorphous systems. Fluorescence imaging revealed enhanced intracellular uptake of both agents. **Conclusions**: This study demonstrates that freeze-dried double-emulsion SEDDS enable efficient co-delivery of hydrophilic and lipophilic drugs, improving stability and cellular uptake. This platform shows strong potential for overcoming key barriers in oral chemotherapy and provides a promising strategy for combination drug delivery.

## 1. Introduction

Lipid-based drug delivery systems (LBDDS) represent a highly versatile platform for enhancing the oral bioavailability of drug molecules. These systems effectively address multiple limitations associated with conventional oral formulations, including poor aqueous solubility, susceptibility to enzymatic degradation in the gastrointestinal (GI) tract, and limited permeability across biological membranes [1]. LBDDS can be broadly classified into three major categories: emulsions, vesicular systems, and lipid particulate systems, each offering distinct physicochemical and biopharmaceutical advantages for oral drug delivery [2,3].

Self-emulsifying drug delivery systems (SEDDS), which belong to the emulsion class of LBDDS, are a particularly functional delivery system for improving drug solubilization and physical stability and can be adapted for delivering lipophilic and hydrophilic molecules [4]. These formulations utilize an oil phase that is dispersed within an aqueous medium (or vice versa) to create stable dispersed systems that enhance the dissolution kinetics of poorly water-soluble drugs [5].

SEDDS are isotropic mixtures typically composed of oils, surfactants, and co-solvents that spontaneously form emulsions upon mild agitation in the aqueous environment [6]. These systems are capable of forming either water-in-oil (w/o) or oil-in-water (o/w) emulsions, depending on the formulation components and processing conditions, thus facilitating the entrapment and delivery of hydrophobic/lipophilic and hydrophilic drug molecules in the internal phase of the emulsion—i.e., aqueous phase of the w/o emulsion and oil phase of the o/w emulsion, respectively [7,8]. Upon dilution with an aqueous medium (e.g., water or gastric physiological solution), SEDDS lipid mixtures self-assemble to form fine dispersions resulting in micro- or nano-emulsions (i.e., SMEDDS or SNEDDS, respectively), depending on the resulting droplet size distribution [9,10].

The pharmacokinetic and therapeutic advantages conferred by SEDDS are wide-ranging, covering a spectrum of delivery routes including the oral route of drug administration. The advantages of SEDDS for oral drug delivery are similarly substantial, and include enhanced drug solubilization and dissolution, leading to improved oral bioavailability; reduced inter- and intra-subject variability in drug absorption; and decreased drug dosages, which may reduce the incidence of adverse drug reactions [6]. Additionally, SEDDS provide a protective lipidic environment that shields drugs from acidic and enzymatic degradation in the GI tract, thereby improving their chemical and metabolic stability for oral route of administration. The ability of these systems to maintain consistent plasma concentration profiles further contributes to enhanced therapeutic efficacy and reduced dosing frequency. Further, the flexibility of SEDDS in accommodating diverse physicochemical properties of drug molecules makes them a highly adaptable platform for oral drug delivery [11,12].

Despite these benefits, liquid SEDDS formulations, as with most dispersed aqueous systems which tend to be intrinsically thermodynamically unstable, often face limitations relating to stability, storage, and dosing accuracy [13,14]. To overcome these challenges for SEDDS containing an aqueous phase as the external phase, freeze-drying (lyophilization) can be employed to convert liquid emulsions into solid dosage forms while preserving the structural integrity and therapeutic viability of the formulation. Freeze-drying is particularly suitable for temperature-sensitive APIs, including proteins, peptides, vaccines, enzymes, and nanoparticles. The process involves the controlled removal of water through sublimation under reduced pressure and low temperature, thereby stabilizing the drug formulation in a dry state suitable for long-term storage.

The lyophilization process comprises three key stages: freezing, primary drying, and secondary drying [15,16]. During freezing, the formulation is solidified to form a matrix of ice crystals, the size and distribution of which determine the porosity of the final dried product. Primary drying involves the sublimation of ice under vacuum, where the product temperature is carefully controlled to prevent collapse or melt-back. Secondary drying removes bound water molecules, reducing the residual moisture content to below 1–5%, which is essential for maintaining the chemical and microbiological stability of the final product [17]. The advantages of freeze-drying include the ability to preserve heat-sensitive materials, enhance the stability of dry powders, facilitate aseptic processing, and produce formulations that rapidly reconstitute upon contact with aqueous media [18].

While freeze-drying is commonly used in converting liquid liposomal formulations which are of a more dilute consistency, the freeze-drying of emulsified systems, which have a relatively higher lipid load, is a more recent area of endeavor in formulation science [19]. Incorporation of a freeze-drying step into SEDDS production yields solid dispersions that are well-suited for fabrication into solid oral dosage forms such as capsules and tablets, while also allowing for reconstitution into suspensions for parenteral administration [6,20]. This approach not only extends the shelf life of the product, but also improves handling, dosing precision, and patient compliance. It thus presents an effective strategy for developing stable, scalable, and patient-friendly oral drug delivery systems [21].

The selection of lipid and surfactant components is critical for achieving efficient drug loading, emulsion stability, and controlled release. In this study, medium-chain triglyceride-based lipids (Labrafac PG) were selected as the oil phase due to their well-established ability to enhance intestinal uptake and solubilize lipophilic drugs [22,23]. Two surfactant systems were comparatively evaluated: Span 80 (sorbitan monooleate) and Caproyl 90 (propylene glycol monocaprylate). Span 80, a low hydrophile–lipophile balance (HLB) surfactant, is commonly used to stabilize primary water-in-oil (w/o) emulsions but often produces larger and less stable droplets [24]. In contrast, Caproyl 90 offers superior solubilization capacity and interfacial activity, promoting the formation of finer emulsions with improved stability [25,26]. This comparative selection was therefore intended to systematically evaluate how surfactant properties influence droplet size, polydispersity, and long-term stability in complex w/o/w systems [27,28].

The rationale for combining doxorubicin and ellipticine lies in their complementary physicochemical properties and therapeutic potential. Doxorubicin is a widely used hydrophilic anthracycline with potent anticancer activity but suffers from poor oral bioavailability and systemic toxicity [29]. Ellipticine, a hydrophobic alkaloid, exhibits strong anticancer effects through DNA intercalation and enzyme inhibition, but is limited by poor aqueous solubility [30]. Co-delivery of these agents offers potential therapeutic synergy, as they act via distinct yet complementary mechanisms of action, potentially enhancing anticancer efficacy while reducing required doses [31]. However, their opposing solubility profiles present a significant formulation challenge, necessitating a system capable of simultaneous encapsulation in spatially distinct compartments.

The current study is a proof-of-principle of a formulation strategy for the concurrent oral delivery of hydrophobic/lipophilic and hydrophilic drug molecules (i.e., dual drug delivery) based on a double-emulsion approach using a water-in-oil-in-water (w/o/w) SEDDS platform. In this system, the hydrophilic drug is encapsulated in the innermost aqueous phase, while the lipophilic drug is entrapped within the intermediate oil phase [32]. The outer aqueous phase acts as a stabilizing medium, ensuring emulsion integrity during gastrointestinal transit. This design facilitates the simultaneous absorption of molecules with disparate solubility profiles while enhancing their stability and bioavailability. The current study outlines the pre-formulation processes involved in fabricating liquid and solid SEDDS, including screening of excipients; followed by the formulation process involving an iterative process technique of optimizing excipient composition, and the biophysical characterization of the resulting SEDDS.

## 2. Materials and Methods

### 2.1. Excipients and Reagents

Labrafac PG (propylene glycol dicaprylate), Labrasol (caprylocaproyl polyoxyl-8 glycerides, NF), Gelucire 44/14 (lauroyl polyoxyl-32 glycerides, NF), and Caproyl 90 (Propylene Glycol Monocaprylate, NF) were obtained from Gattefossé Corporation (Paramus, NJ, USA). Span 80 was obtained from Thermo Fisher Scientific (Waltham, MA, USA), Sucrose was obtained from Spectrum Chemicals (New Brunswick, NJ, USA) and Vectashield Antifade Mounting Medium with DAPI was obtained from Vector Laboratories Inc. (Newark, CA, USA). All the chemicals used for experiments were of reagent grade. MDA-MB-468 (ATCC HTB-132) and MDA-MB-231 (ATCC HTB-26) cells were purchased from the American Type Culture Collection (ATCC, Manassas, VA, USA). MDA-MB-468 and MDA-MB-231 cells were maintained in DMEM/F12 media supplemented with 10% FBS and a 2% mixture of penicillin, streptomycin, and neomycin (PSN), all of which were obtained from Thermo Fisher Scientific (Waltham, MA, USA), at 37 °C in a humidified incubator with 95% air and 5% CO_2_.

### 2.2. Excipient Screening and Preformulation

The excipients used for this screening are all Food and Drug Administration (FDA)-approved with varying HLB values and required HLB (RHLB), comprising Labrafac PG (oil, HLB 1), Labrasol (surfactant, HLB 12), Caproyl 90 (co-surfactant, HLB 2), Gelucire 44/14 (co-surfactant, HLB 11) and Span 80 (surfactant, HLB 4.3). Different proportions of each excipient were used based on their HLB and RHLB in determining their facilitation of formation of primary and double emulsions.

### 2.3. Ternary Phase Diagram

Self-emulsifying drug delivery systems (SEDDS) were developed using ternary phase diagrams constructed with an online two-dimensional graphing tool from https://sanplot.com/shiny/triangle/ (accessed on 24 July 2025). These diagrams were used to determine the appropriate concentration ranges of the oil phase and surfactant mixture required to facilitate spontaneous emulsification upon dilution with water. Various lipid mixtures composed of Labrafac PG, Labrasol, Gelucire 44/14, Span 80, and Caproyl 90 were investigated. The influence of these excipients on self-emulsification behavior was evaluated by constructing ternary phase diagrams in which Labrafac PG was used as the oil phase, Labrasol as the surfactant, and Gelucire 44/14 together with Span 80 as co-surfactants. The interaction of these components with double-distilled water was assessed to identify the self-emulsifying region within the diagram. The relative proportions of the three components (i.e., oil, surfactant/co-surfactant mixture, and aqueous medium) were represented within an equilateral triangular coordinate system, where each vertex corresponds to 100% of a single component and each side represents the compositional variation between the components [33,34].

### 2.4. Preparation and Optimization of Self-Emulsifying Drug Delivery System (SEDDS)

**Figure 1:**
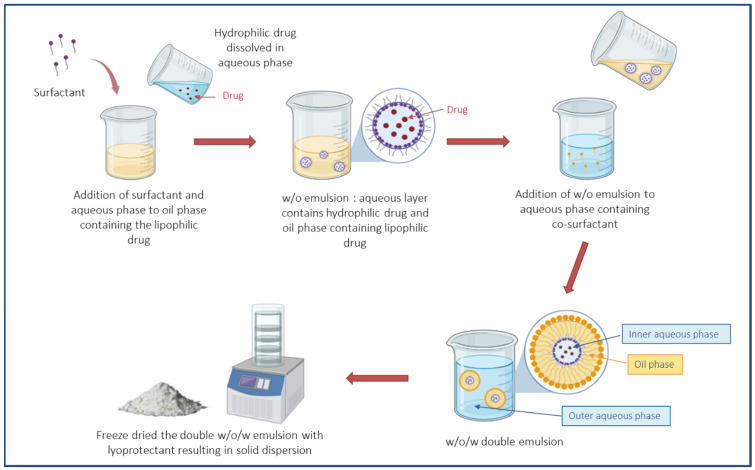
Method of preparation of freeze-dried double-emulsion SEDDS, Created in BioRender. Soni, S. (2026) https://BioRender.com/u4yftpk (accessed on 21 March 2026).

**Figure 2:**
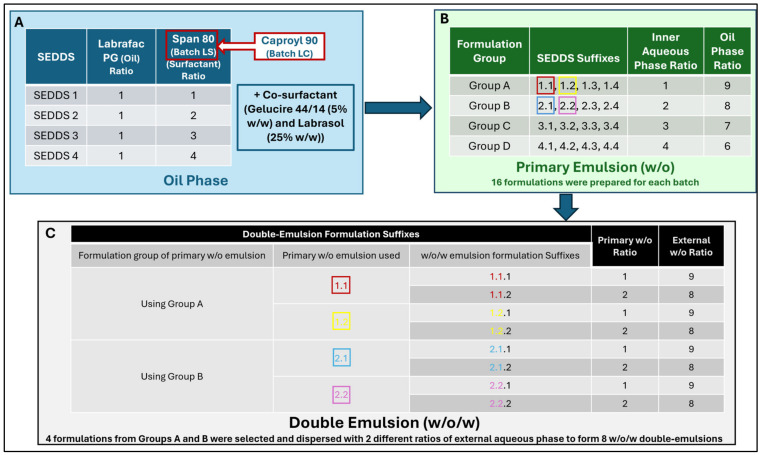
Schematic representation of optimization of a two-stage emulsification process leading to the formation of a self-emulsifying drug delivery system (SEDDS). (**A**) Excipient optimization process involving determining of the relative ratios of surfactants (Span 80; Capryol 90) to be combined with the oil mixture (Labrafac PG, Labrasol, and Gelucire 44/14) to form the oil phase. Four SEDDS formulations were prepared using varying ratios of each Labrafac PG with either Span 80 (batch LS) or Caproyl 90 (batch LC), each containing 5% *w*/*w* Gelucire 44/14 and 25% *w*/*w* Labrasol, resulting in a total of eight oil-phase systems (SEDDS). (**B**) The resulting oil-phase systems were dispersed at four different ratios of internal aqueous phase to produce sixteen primary water-in-oil (w/o) emulsions for each batch (LS and LC), yielding thirty-two primary w/o formulations overall. (**C**) Two primary emulsion (w/o) formulation groups with better stability profiles (i.e., Groups A and B) were selected and dispersed in an external aqueous phase at two different ratios to obtain eight double-emulsion (w/o/w) systems.

The self-emulsifying drug delivery system (SEDDS) was prepared as previously described with some modifications [35]. The formulation consisted of Labrafac PG as the oil excipient, Labrasol and Span 80 as surfactants, and Caproyl 90 and Gelucire 44/14 as co-surfactants. Various ratios of the excipients were evaluated for the formation of stable self-emulsifying formulations. Drug-free (Blank) SEDDS were prepared, starting with the oil phase, which was prepared by mixing the oil excipient with the selected surfactants and co-surfactants under continuous stirring until a homogeneous mixture was obtained. The aqueous phase—distilled water—was then gradually incorporated into the oil–surfactant mixture under gentle agitation to form a primary water-in-oil (w/o) emulsion. Subsequently, an external aqueous bulk phase comprising sucrose solution in distilled water was prepared with the sucrose serving as a lyoprotectant in a subsequent freeze-drying step. The prepared w/o emulsion, acting as the internal phase, was slowly added to the aqueous phase (sucrose solution) under continuous gentle agitation to obtain a water-in-oil-in-water (w/o/w) double-emulsion system. The emulsification process was carried out at room temperature (25 °C) to ensure uniform droplet formation and emulsion stability [36]. The resulting double-emulsion was subsequently subjected to freeze-drying (lyophilization) to remove the aqueous phase and obtain a dried solid dispersible product. Figure 1 represents the schematic diagram of each step from formulating the SEEDS preparation to forming solid dispersion final product. Figure 2 represents the schematic diagram of different ratios of each phase for optimizing the preparation. In loading drug molecules into SEDDS, where applicable, hydrophilic drug molecules were dispersed in the internal aqueous phase of the primary w/o emulsion whereas lipophilic drug molecules were added to the (intermediate) oil phase prior to the emulsification process; doxorubicin and ellipticine were utilized as model hydrophilic and lipophilic drugs, respectively [37]. While diametrically opposite in their polar behavior, doxorubicin and ellipticine both exhibit intrinsic fluorescence behavior, which lends itself to imaging for assessing cellular drug uptake.

### 2.5. Freeze-Drying of Liquid SEDDS to Produce Solid Dispersible SEDDS

The freeze-drying of the emulsions was conducted using a Labconco FreeZone Legacy 2.5 Liter Benchtop Freeze Dryer (Fisher Science Education, Hanover Park, IL, USA) with a cycle duration of approximately 10 to 24 h, where the samples were frozen in −80 °C freezer for 12 h and then hooked up to the freeze-dryer [38,39].

### 2.6. Stability Determination of Primary and Double-Emulsion

Stability of the emulsions was evaluated by visual observation of the formation of stable emulsions without phase separation. All formulations were monitored daily at room temperature (25 °C) over a 2-week period and assessed for phase separation as well as agglomeration by measuring changes in droplet size at the end of that period. In addition, variations in droplet size and polydispersity index (PDI) among the different formulations were analyzed and compared.

### 2.7. Characterization for Droplet Size and PDI

Droplet size, polydispersity index (PDI), and zeta potential of undiluted liquid SEDDS and dispersions of solid SEDDS in demineralized water (0.33, 1.67 and 3.33% *w*/*v*) were performed using 90 Plus Particle Size Analyzer (Brookhaven Instruments Corporation, Nashua, NH, USA) [40]. Approximately 3 mL of SEDDS were placed in the cuvette followed by dynamic light scattering measurements for droplet size and PDI, and zeta potential measurement using a palladium electrode-equipped dip cell [41]. The concentration of the undiluted SEDDS preparation was in the range of 10–20% *v*/*v* for all formulations. All measurements were performed at least three times on triplicates of each formulation batch. Freeze-dried SEDDS were reconstituted in an aqueous phase by dispersing 10 mg, 50 mg, and 100 mg of the solid-dispersible SEDDS in 3 mL distilled water at final concentrations of 0.33, 1.67 and 3.33% *w*/*v*, respectively. The reconstituted formulations were analyzed for droplet size and PDI using 90 Plus Particle Size Analyzer (Brookhaven Instruments Corporation).

### 2.8. Validation of Double-Emulsion (w/o/w) Formation

The liquid emulsion system was assessed for formation of a w/o/w double-emulsion using a modified dye staining protocol with trypan blue—a hydrophilic dye. Trypan blue easily dissolves in an aqueous medium and undergoes partitioning into the aqueous phase of a binary mixture of oil and water. In this experiment, 100 µL of 0.4% *w*/*v* trypan blue dye was added to a 1 mL formulation and incubated at room temperature for 10 min under gentle agitation. A small amount of the formulation was placed on a glass slide using a dropper and three layers of depth with a Z-stack of micrograph images acquired under brightfield light using an EVOS M7000 Imaging System (Thermo Fisher Scientific, Waltham, MA, USA). Double-emulsion formation was verified by capturing micrographs the partitioning of the trypan blue dye within the inner aqueous phase of the w/o emulsion and the external/continuous aqueous phase of the w/o/w, with the intermediate oil phase delineating the two aqueous phases as a yellowish-brown lamellar.

### 2.9. Differential Scanning Calorimetry (DSC) Analysis

Differential scanning calorimetry (DSC) was performed using a TA Instrument Q20 DSC (Waters Corporation, Milford, MA, USA). Samples (approximately 10 mg) were loaded into Tzero aluminum pans, ensuring flat placement, and hermetically sealed using a Tzero press with blue die sets (Waters Corporation, Milford, MA, USA) to achieve high-pressure seals (2–3 atm) and optimal thermal contact. A matched, empty sealed Tzero pan was used as the reference. The sealed pans were then placed in the Q20 DSC cell, and thermal scans were conducted between −20 °C and 250 °C [42]. Results were presented as thermograms of heat flow against temperature.

### 2.10. Drug Encapsulation Efficiency

Doxorubicin and ellipticine entrapment efficiency of the liquid SEDDS were determined on the basis of the amount of internalized drug as a ratio of solid SEDDS weight using the following formula:

$$Entrapment\;Efficiency\left(\%\right)=\frac{Total\;drug\;added-Free\;drug\;released}{Total\;drug\;added}\times 100$$

In determining the total amount of drug in the SEDDS formulation, approximately 500 µL of the SEDDS formulation was placed in a microcentrifuge. The sample was vortexed and then centrifuged at 13,000 rpm for 5 min. The supernatant containing free drug was collected and run through HPLC analysis to quantify the free drug. The experiment was carried out in triplicates for each formulation and results were computed as means ± SD.

The HPLC analysis was performed using a mobile phase consisting of 60% acetonitrile as the organic phase and 40% 10 mM disodium hydrogen phosphate (Na_2_HPO_4_, pH 3.5) as the aqueous phase (prepared using HPLC grade water). Chromatographic separation was performed on a Phenomenex Luna C18 column (5 µm particle size) (Phenomenex, Torrance, CA, USA) under isocratic conditions.

The analysis was carried out using an Alliance Water 2695 HPLC system (Waters Corporation, Milford, MA, USA) at a flow rate of 0.8 mL/min, with a total run time of 12 min. Detection was performed at a wavelength of 240 nm using a UV–Vis detector. Data acquisition and analysis were conducted using Empower 3 software.

### 2.11. Cellular Uptake Assay

For the cellular uptake study, the drugs were incorporated into the formulation and analyzed for their uptake by cells. Two drugs were loaded in one of these prepared SEDDS to see that this prepared formulation could be served as a proof of concept for dual drug delivery. The first drug, doxorubicin, is a hydrophilic compound with red fluorescence and was loaded into the internal aqueous phase of the w/o/w double emulsion formulation. The second drug, ellipticine, is a lipophilic compound with green fluorescence and was loaded into the oil phase of the w/o/w double emulsion formulation.

MDA-MB-468 and MDA-MB-231 (triple-negative breast cancer cell-lines) cells were seeded at 1 × 10^4^ cells/well in a 96-well format using EZsphere plates (AMSBIO LLC, Cambridge, MA, USA) and incubated at 37 °C for 5 days to facilitate spheroid formation. Cells were then exposed to formulations containing doxorubicin and ellipticine for 30 min, followed by staining with DAPI (4′,6-diamidino-2-phenylindole) to visualize nuclei. The SEDDS formulations were then aspirated; the cells were washed twice with PBS and imaged using an EVOS M700 Imaging System under red, green, and blue fluorescence filters (Thermo Fisher Scientific, Waltham, MA, USA).

## 3. Results

### 3.1. Excipient Screening and Preformulation

Oil (Labrafac PG), surfactant (Labrasol, Caproyl 90), and co-surfactant (Span 80, Gelucire 44/14) excipients were mixed at different ratios and observed for miscibility of the oil/surfactant/co-surfactant mixture, and when dispersed in water, resulting in four (4) oil/surfactant/co-surfactant mixture types/phases (i.e., SEDDS 1–4). When each of the four oil phases was dispersed in water (aqueous phase), relatively homogenous liquid dispersions were observed at weight ratios ranging from 1:9 to 5:5 of oil phase to aqueous phase, with gel formation observed at ratios of 6:4 to 9:1 of oil phase to aqueous phase (Table 1). This gel formation can be attributed to enhanced interaction between surfactants, co-surfactant, oil, and water, leading to the formation of structured mesophases such as liquid crystalline or discontinuous systems. While moderate gel formation may enhance stability, excessive gelation can hinder self-emulsification by slowing dispersion and reducing the spontaneity of emulsion formation. Thus, the combinations of oil and surfactant/co-surfactants resulting in liquid dispersions were subsequently used in further identifying stable emulsion formation using Ternary phase diagrams.

### 3.2. Ternary Phase Diagram

**Figure 3:**
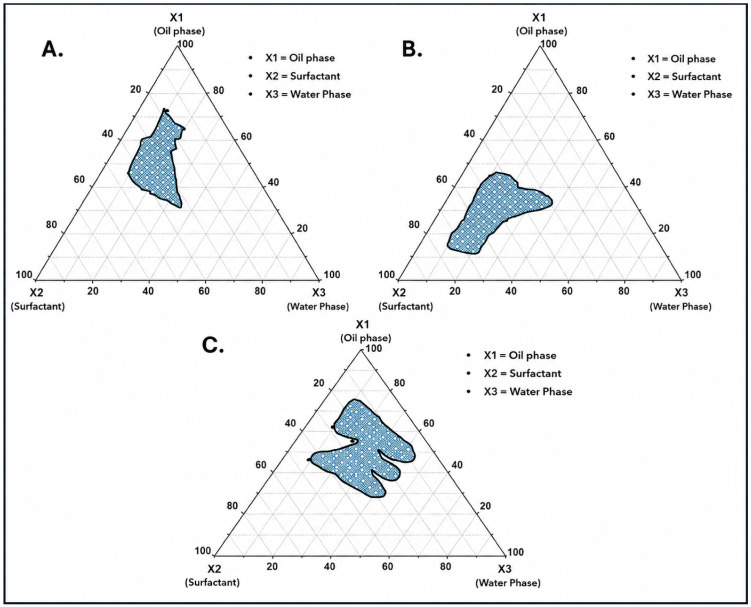
Ternary phase diagrams showing regions of stable emulsification formed from combinations of oil (Labrafac PG), surfactant/co-surfactant (Labrasol and Gelucire 44/14/Capryol), and water. (**A**) Ternary phase diagram resulting in water-in-oil (w/o) primary emulsion formation of dilutions of oil phase and aqueous phase within the ratio range of 1:9 to 5:5, respectively, based on Table 1. (**B**) Ternary phase diagram of water-in-oil (w/o) primary emulsion of dilutions of oil phase and aqueous phase within the ratio range of 6:4 to 9:10. (**C**) Ternary phase diagram of water-in-oil-in-water (w/o/w) double-emulsion formation of dilutions of water-in-oil (w/o) primary emulsion composition from (**B**) dispersed in an aqueous continuous phase within the ratio range of 1:9 to 5:5. The shaded region within the triangle represents all combinations of oil, aqueous phase, and emulsifier (surfactant/co-surfactant) concentrations that result in stable emulsions. The region outside the triangle represents combinations that do not result in stable emulsions.

Ternary phase diagrams were generated with different ratios of oil, surfactant/co-surfactant, and aqueous phase. The textured area in the ternary plot (Figure 3) indicates a stable formation of emulsion, which was relied on for the optimization of the oil phase and aqueous mixture for forming the primary emulsions, and the subsequent formation of the double emulsions.

### 3.3. Optimization of Oil–Surfactant Mixtures

By using the ternary phase plot, various oils and surfactants were screened to assess their compatibility as oil–surfactant mixtures. Mixtures of Labrafac PG and Span 80 were evaluated at four different oil-to-surfactant ratios: 1:1, 1:2, 1:3, and 1:4, to optimize the concentration of the oil–surfactant blend (Table 2, Batch LS). A second batch of mixtures (Table 2, Batch LC), comprising Labrafac PG and Caproyl 90, were also prepared and evaluated at the same oil-to-surfactant ratios as Batch LS.

### 3.4. Optimization of Different Ratios of w/o Primary Emulsion

For each SEDDS oil mixture listed in Table 2, four distinct aqueous phase–to–oil phase ratios were evaluated to optimize the formation of stable water-in-oil (w/o) emulsions. The oil phase, consisting of surfactant, co-surfactant, and oil, was first homogenized, followed by the gradual addition of the aqueous phase under gentle stirring to obtain the w/o primary emulsion.

The aqueous-to-oil phase ratios investigated were 1:9, 2:8, 3:7, and 4:6 (*w*/*w*), corresponding to formulation groups A, B, C, and D, respectively. These ratios corresponded to weight compositions (mg) of 100:943 (formulation group A), 200:838 (formulation group B), 300:733 (formulation group C), and 400:628 (formulation group D); see Table 3. A total of 32 SEDDS formulations were prepared across Batches LS and LC. All formulations were subsequently evaluated for stability and droplet size. Based on these assessments, 16 formulations demonstrating acceptable stability were shortlisted, primarily from formulation groups A and B (8 each from Batches LS and LC), which exhibited superior emulsion stability compared to the other ratios.

However, formulations containing Span 80 (Batch LS) were excluded due to their relatively high viscosity, which resulted in larger droplet sizes than desired. Thus, formulations containing Caproyl 90 (Batch LC) were selected due to their ability to yield stable and homogenously dispersed emulsions with comparatively smaller and more acceptable droplet sizes. Consequently, eight (8) Batch LC formulations were selected for further evaluation.

Stability studies conducted over 24 h indicated that formulations with lower aqueous phase content (i.e., higher oil phase proportion) resulting from SEDDS 1 and 2 exhibited improved emulsion stability. Specifically, formulation groups A (1:9) and B (2:8), with SEDDS 1 and 2 compositions, demonstrate the most stable primary emulsions. Based on these findings, a total of four formulations each from these ratios were selected for further investigation. This w/o primary emulsion served as internal phases for formation of the w/o/w double-emulsion. See Figure 2 for a schematic explanation of this primary w/o emulsion optimization process.

### 3.5. Optimization of (w/o/w) Double-Emulsion

In formulating the water-in-oil-in-water (w/o/w) double-emulsion, the selected w/o primary emulsions (formulation groups A and B; 4 SEDDS) served as the internal phase, while the external aqueous phase consisted of sucrose dissolved in distilled water, with sucrose acting as a lyoprotectant for a subsequent freeze-drying step. Two different internal-to-external phase ratios were investigated to optimize the formation of double-emulsion. Specifically, formulation A1.1–A1.4 employed a 1:9 ratio of internal phase (w/o primary emulsion) to external aqueous phase, whereas formulation B2.1–B2.4 employed a 2:8 ratio, corresponding to weight ratios (mg) of 0.1:0.943 (formulation A1.1–A1.4) and 0.2:838 (formulation B1.1–B1.4), respectively. For example, for SEDDS 1 in Batch A (Table 2), formulation A1.1 to A1.4 corresponded to w/o to external aqueous phase weight ratios of 0.1:0.943 for SEDDS 1 to 4, respectively. In all, there were eight (8) SEDDS formulations obtained using SEDDS 1 and 2 from batches B-LC. The composition of the double emulsion formulations for each component with the formulation number are shown in Table 4.

### 3.6. Stability Determination of Primary (w/o) and Double (w/o/w) Emulsion:

**Figure 4:**
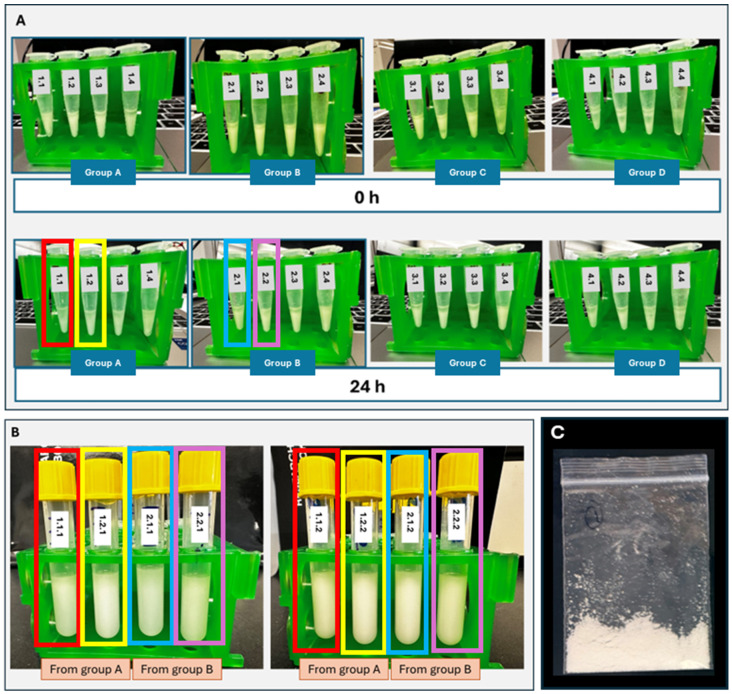
(**A**) Stable primary water-in-oil (w/o) emulsions. (**B**) Stable water-in-oil-in-water (w/o/w) double-emulsions after 24 h. (**C**) Freeze-dried SEDDS. Formulations labeled 1.1, 1.2, 1.3, and 1.4 represent different oil-to-surfactant ratios used to prepare the primary w/o emulsions for formulation group A (1:9 ratio of water:oil), and 2.1, 2.2, 2.3 and 2.4 represent those for formulation group B (2:8 ratio of water:oil). The same label convention applies for formulation group C (3:7 ratio of water:oil) and formulation group D (4:6 ratio of water:oil). The double-emulsions were subsequently prepared from these primary emulsions. Formulations 1.1.1, 1.2.1, 2.1.1, and 2.2.1 correspond to a 1:9 internal (primary w/o emulsion) phase to external aqueous phase ratio, while formulations 1.1.2, 1.2.2, 2.1.2, and 2.2.2 correspond to a 2:8 ratio (primary w/o emulsion:external aqueous phase) for formation of double-emulsions.

Visual assessment of the emulsions for stability showed that the primary emulsions with ratios of 1:9 and 2:8 of internal aqueous phase to oil phase yielded stable w/o primary emulsions. Subsequently, the two different ratios of the internal w/o to external aqueous phases, 1:9 and 2:8, for w/o/w double-emulsion, resulted in stable systems. Visually, there was no phase separation between two phases for these emulsions, and there was no change in droplet size over 24 h (Figure 4A,B).

### 3.7. Optimization of Freeze Drying and Using Flash Freezing

Freezing of samples was achieved by storing the samples in −80 °C for 12 h followed by freeze-drying for 18 h to obtain a solid-dispersible final product. A representative image of the resulting freeze-dried product is shown in Figure 4C.

### 3.8. Droplet Size Analysis, Zeta Potential and Polydispersity Index (PDI)

The droplet size for primary (w/o) emulsion for the Batch LS was observed in the range of 2115 ± 0.51 nm and 655 ± 0.08 nm (Table 5). The Batch LS (Labrafac PG-Span 80) formulations had a viscous consistency with relatively larger heterogenous droplets with lower stability compared to Batch LC (Labrafac PG-Caproyl 90) which exhibited smaller droplet and homogenous size ranges (575.2 ± 46.1 nm and 89.65 ± 1.55 nm).

After 28 days of storage, the double-emulsion systems exhibited clear differences in stability depending on the surfactant used as shown in Table 6. Formulations prepared with Span 80 (Batch LS, Table 5 and Table 6) showed a marked increase in droplet size, ranging from approximately 2.5 µm to 7.6 µm, accompanied by higher polydispersity index (PDI) values (0.234–0.954). This substantial growth in droplet size indicates pronounced coalescence and instability within the system over time. The elevated PDI values, particularly those approaching 1.0, reflect highly heterogeneous dispersions with broad size distributions. Among the formulations, 1.1.2 and 2.1.2 were especially unstable, exhibiting both the largest droplet sizes and highest PDI values. Even formulations with comparatively lower PDI, such as 1.1.1, still display micro-sized droplets, which are considered unsuitable for stable emulsion systems. Overall, these findings suggest that Span 80 will be insufficient for maintaining long-term stability in w/o/w double emulsions, likely due to higher viscosity, leading to droplet aggregation and eventual phase separation.

In contrast, formulations containing Caproyl 90 (Batch LC—Table 5 and Table 6) demonstrated significantly improved stability after 28 days. Droplet sizes remained within the nano to submicron range (approximately 213–765 nm), with low to moderate PDI values (0.015–0.576). Several formulations, particularly 1.2.1 and 2.2.1, exhibited very low PDI values (<0.05), indicating highly uniform and monodisperse systems. Compared to Batch LS, these formulations showed minimal droplet growth over time, suggesting strong resistance to coalescence and improved interfacial stabilization. Although some variability was observed in certain formulations (e.g., 2.1.2), the overall size distribution and stability remained significantly superior to the Span 80-based systems. These results confirm that Caproyl 90 provides a more effective stabilizing environment for w/o/w emulsions, maintaining both droplet size and homogeneity over extended storage.

Additionally, when compared with the initial primary w/o emulsions, the instability of Batch LS becomes even more evident. The primary w/o emulsions exhibited droplet sizes in the range of 655 ± 0.08 nm to 2115 ± 0.51 nm; however, after conversion to w/o/w systems and 28 days of storage, droplet sizes increased dramatically to 2547–7645 nm. This 2–3-fold increase (or greater) indicates severe coalescence and possible disruption of the internal aqueous phase, supporting the conclusion that Batch LS formulations are inherently unstable and prone to structural breakdown. In contrast, Batch LC showed only a moderate increase in droplet size, from an initial range of 89.65 ± 1.55 nm to 575.2 ± 46.1 nm in the primary w/o emulsions to 213–765 nm after 28 days in the w/o/w systems. Importantly, these values remain within an acceptable nano-sized range, indicating that the structural integrity of the emulsion was preserved. This behavior reflects improved interfacial stabilization and resistance to droplet coalescence, further reinforcing the suitability of Caproyl 90 for developing stable double-emulsion-based delivery systems.

The droplet size of the freeze-dried solid dispersion was analyzed for three different dilution ratios of 0.33, 1.67 and 3.33% *w*/*v* and found to be 937.2 ± 13.2 nm, 867.6 ± 63.4 nm and 717.7± 39.1 nm, respectively. The PDI values of ratios of 0.33, 1.67 and 3.33% *w*/*v* were found to be 0.335 ± 0.005, 0.150 ± 0.023 and 0.094 ± 0.015.

### 3.9. Validation of w/o/w Double-Emulsion Formation

**Figure 5:**
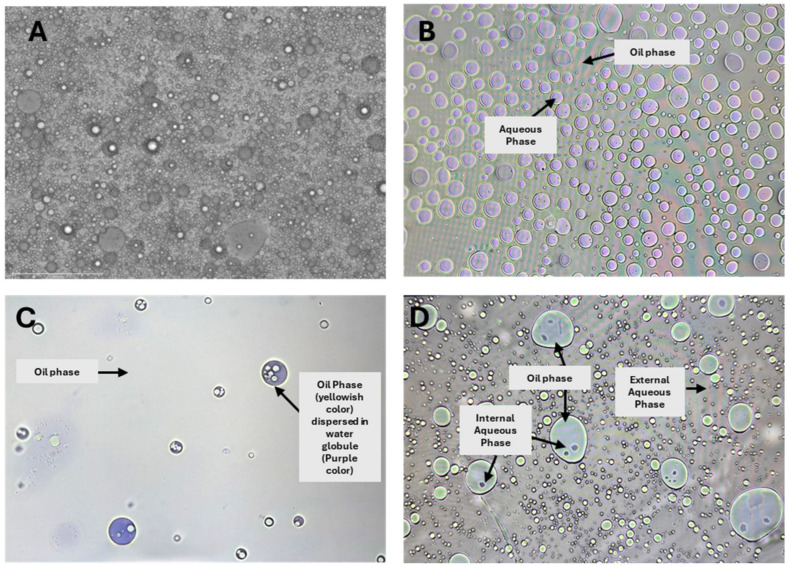
Emulsion formulation under normal brightfield light using an EVOS M700 Imaging System showing microscopic images of aqueous oil dispersions with and without staining with hydrophilic trypan blue dye. (**A**) Micrograph of an unstained w/o/w emulsion showing no color differentiation of dispersed vs. continuous phase. (**B**) Micrograph showing trypan blue staining of a w/o primary emulsion showing blue-stained aqueous droplets (internal phase) dispersed in an oil continuous phase. (**C**) Micrograph showing an oil-in-water-in-oil (o/w/o) double-emulsion with an unstained inner oil phase within a stained dispersed phase within an unstained continuous oil phase. (**D**) Micrograph showing a water-in-oil-in-water (w/o/w) double-emulsion with a stained inner aqueous phase within an unstained dispersed oil phase within a continuous aqueous phase. The blue-to-purple staining is from the trypan blue dye, and the yellowish color is the natural color from the oil phase.

The image in Figure 5B showed the internalization of the blue-stained aqueous phase in the yellow oil phase for the primary emulsion formation. This primary emulsion was then again dispersed into the external aqueous phase to form a double-emulsion as shown in Figure 5D. Here are pictures of different emulsion formations in Figure 5.

### 3.10. Differential Canning Calorimetry (DSC) Analysis

**Figure 6:**
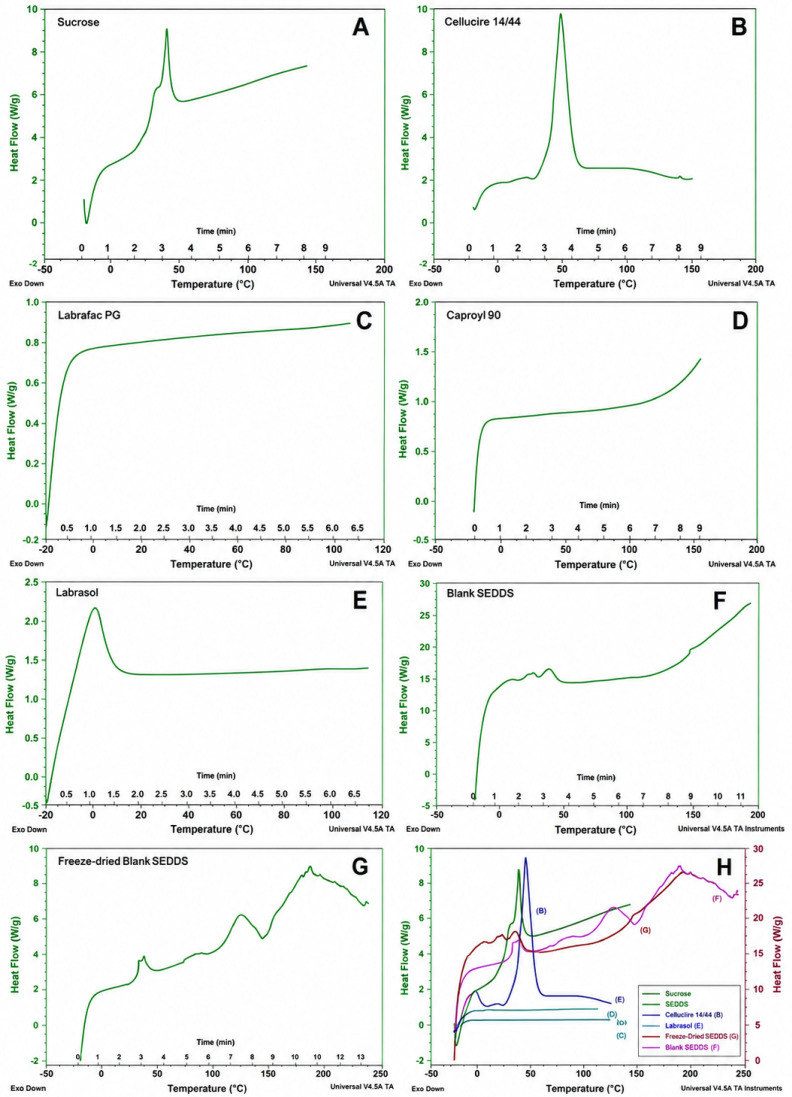
Thermograms of individual excipients, formulated SEDDS, and merged plots. (**A**) Sucrose; (**B**) Gelucire 44/14; (**C**) Labrafac PG; (**D**) Caproyl 90; (**E**) Labrasol; (**F**) Liquid w/o/w Blank SEDDS; (**G**) Freeze-Dried Blank SEDDS; and (**H**) Composite thermograms (**A**–**H**) super-imposed on each other. The thermograms were derived from thermal analysis using a TA Instrument Q20 DSC.

Differential scanning calorimetry (DSC) was performed to evaluate the thermal behavior, crystallinity, and possible interactions among formulation components and the formulated SEDDS preparations. The thermograms of individual excipients and the formulated SEDDS are presented in Figure 6. The thermogram obtained of sucrose exhibits a characteristic of endothermic peak in the range of approximately 40.23 °C, corresponding to its melting or dehydration, confirming its crystalline nature (Figure 6A). In contrast, Gelucire 44/14 showed a sharp endothermic peak around 45.32 °C which is attributed to its melting point and indicates its semi-crystalline lipidic structure (Figure 6B). The thermogram of Labrafac PG, Caproyl 90 displayed a broad and low-intensity thermal event without a distinct melting peak, consistent with its liquid and amorphous nature (Figure 6C,D), with Labrasol showing a peak at −0.68 °C that remains nominally unchanged from baseline (Figure 6E).

The liquid Blank SEDDS formulation exhibited altered thermal characteristics compared to the individual components (Figure 6F). The melting peak of Gelucire 44/14 was reduced intensity and slightly shifted, suggesting improved miscibility and possible interactions among the formulation components. Additionally, the absence of sharp, well-defined peaks indicates a reduction in crystallinity and the formation of a more homogeneous system.

Freeze-dried SEDDS (Figure 6G) demonstrated significant changes in its thermal profile. The characteristic peaks of both Gelucire 44/14 and sucrose were broadened and diminished, indicating a transition from crystalline to an amorphous or partially amorphous state following lyophilization. The overall thermogram displayed broad transitions rather than distinct melting peaks, confirming the formation of an amorphous solid dispersion. Super-imposed thermograms in Figure 6H highlight the relative variations in thermal behavior as well as areas of convergence at certain temperature regions, particularly with liquid and freeze-dried SEDDS.

### 3.11. Drug Entrapment Efficiency

The entrapment efficiencies of ellipticine- and doxorubicin-loaded SEDDS formulation was evaluated, and the results showed 98.17 ± 0.91% of doxorubicin and 97.25 ± 0.84% of ellipticine drug was entrapped in the formulation. Both drugs demonstrated high entrapment efficiency (>97%), confirming the drug solubilization in the system and effectiveness of SEDDS as a drug delivery system.

### 3.12. Cellular Uptake

**Figure 7:**
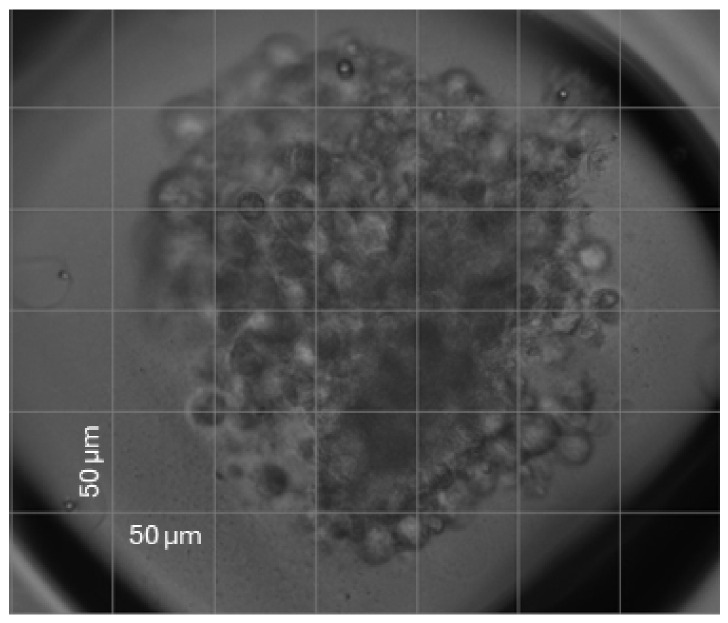
Microscopic image of the spheroid formation at day 5 shown at 20× magnification with a grid size of (50 × 50) µm size.

**Figure 8:**
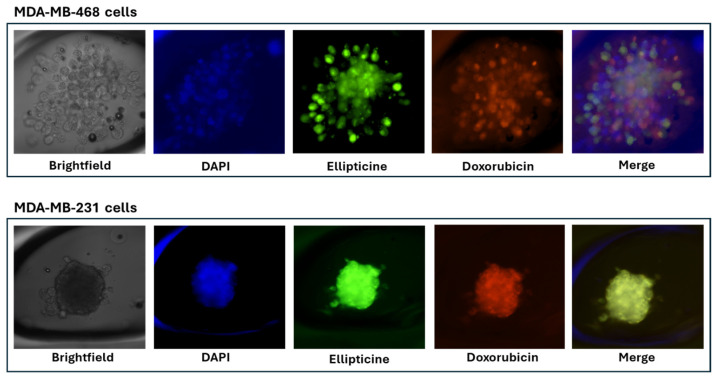
Fluorescence micrograph (20× magnification) of doxorubicin and ellipticine-loaded SEDDS uptake in MDA-MB-468 and MDA-MB-231 cells. The cells were exposed to dual drug-loaded SEDDS for 30 min and stained with DAPI (blue) nuclear dye to allow for the visualization of the internalization and co-localization of doxorubicin (red) and ellipticine (green) within the cell. The overlap of red, green, and blue signals in the merged image indicates the localization of doxorubicin and ellipticine-loaded SEDDS.

The cellular internalization of doxorubicin- and ellipticine-loaded liquid SEDDS in a three-dimensional (3D) spheroid model of breast cancer was investigated by fluorescence microscopy using an EVOS M700 Imaging System under red (ellipticine), green (doxorubicin), and blue (DAPI) fluorescence filters. The microscopic image of the spheroid formed is observed in Figure 7; the image is 20× magnification for the system and the grid represents (50 × 50) µm size. MDA-MB-468 and MDA-MB-231 spheroid cell cultures were exposed to dual-drug loaded liquid SEDDS, and the fluorescence was assessed (Figure 8). The merged image showed internalization of the SEDDS formulation and co-localization of doxorubicin and ellipticine in the nucleus (blue staining).

## 4. Discussion

Vesicular systems, including liposomes, noisomes, transferosomes, and exosomes, have been extensively explored as lipid-based carriers capable of encapsulating active pharmaceutical ingredients (APIs) within lipid bilayers, thereby offering protection against enzymatic and acidic degradation in the gastrointestinal tract. This structural advantage also promotes enhanced drug transport across intestinal epithelial barrier through endocytic or transcellular pathways [43]. In parallel, lipid particulate systems, such as lipospheres, solid lipid nanoparticles (SLNs), lipid-drug conjugates, and nanostructured lipid carriers (NLCS), offer additional benefits including controlled drug release, improved formulation stability, and enhanced bioavailability, particularly for compounds with poor aqueous solubility [44].

Lipid-based drug delivery systems offer numerous advantages over traditional drug formulation approaches. These include improved physicochemical stability, which ensures the retention of drug potency over extended storage durations; and high drug loading capacity, allowing for efficient encapsulation of both hydrophilic and lipophilic drugs. Additionally, the use of biodegradable and biocompatible lipid excipients minimizes the potential for immunogenic or toxic reactions [45]. Furthermore, the flexibility in selecting excipients and formulation techniques enables the development of customized delivery platforms tailored to specific pharmacokinetic and therapeutic requirements. Passive, non-invasive vesicular systems, such as liposomes and niosomes, demonstrate favorable scalability and commercialization potential, thereby facilitating the translation of laboratory-scale innovations into clinically viable products [46,47].

Among these lipid-based approaches, self-emulsifying drug delivery systems (SEDDS) have emerged as a particularly promising platform due to their ability to significantly enhance the oral absorption of hydrophobic drugs by promoting spontaneous emulsification in gastrointestinal (GI) fluids [48]. However, despite their advantages, the formulation of SEDDS, particularly those involving double-emulsions, presents notable challenges. One of the primary formulation challenges in developing self-emulsifying drug delivery systems (SEDDS) was achieving a stable primary w/o emulsion, which serves as the foundation for the subsequent w/o/w double-emulsion structure. Formulating a robust w/o/w emulsion requires careful selection and optimization of oils, surfactants, and co-surfactants, along with precise control over phase ratios [6].

To address these challenges, a systematic proof-of-principle formulation strategy for dual drug delivery was employed using liquid self-emulsifying drug delivery systems (SEDDS) and subsequently fabricated into solid-dispersible dosage forms via freeze-drying for various potential applications including oral delivery. Initial excipient screening evaluated the miscibility of different combinations of oils, surfactants, and aqueous phases. Subsequently, ternary phase diagrams were constructed to delineate the optimal compositional regions for primary—two-phase, w/o—emulsion and secondary—three-phase, w/o/w—emulsion formation [6]. These phase diagrams guided the selection of appropriate oil-to-surfactant ratios, facilitating the formulation of stable primary w/o emulsions and their further emulsification into stable w/o/w double emulsion systems [49].

Ternary phase diagrams illustrate the interactions among three key components: oil, surfactant, and water. In SEDDS preparation, these diagrams enable the identification of optimal formulations that promote stable water-in-oil (w/o) emulsions. The oil phase serves as the solvent for lipophilic drugs, while surfactants reduce interfacial tension, facilitating emulsion stability. The aqueous phase, typically composed of water or aqueous solvents, is essential for creating the final emulsion [50]. Constructing a ternary phase diagram involves systematically varying the proportions of these components and assessing their phase behavior, thereby mapping stable and unstable regions within the diagram. This graphical representation not only aids in optimizing formulation ratios but also predicts emulsion stability under different conditions, which is vital for ensuring product performance. Ultimately, the ternary phase diagrams showed regions of stable emulsion formation and guided the approach toward formulating both liquid and solid-dispersible SEDDS [51].

Initial excipient screening demonstrated that combinations of Labrafac PG with Labrasol, Gelucire 44/14, and either Span 80 or Caproyl 90 produced homogenous liquid dispersions at oil phase-to-aqueous phase ratios ranging from 1:9 to 5:5. However, gel formation observed at higher oil phase-to-aqueous phase ratios (6:4 to 9:1) is likely attributable to the formation of surfactant-rich lyotropic liquid crystalline mesophases resulting from increased intermolecular organization between the lipid components, surfactants, co-surfactants, and limited aqueous content. At elevated oil/surfactant concentrations, insufficient water is available to fully hydrate the surfactant head groups, promoting ordered interfacial packing and transition from isotropic nanoemulsion systems to more viscous bi-continuous, hexagonal, or lamellar structures. Such phase transitions are commonly reported in lipid-based self-emulsifying systems containing nonionic surfactants and medium-chain lipids [52]. In particular, Gelucire 44/14 and Span 80 are known to contribute to structured interfacial assemblies due to their amphiphilic characteristics and low hydrophile–lipophile balance (HLB), which may further enhance viscosity and mesophase formation at reduced aqueous content.

While moderate structuring can improve thermodynamic stability by reducing droplet coalescence, excessive gelation may impair spontaneous emulsification by slowing dispersion kinetics and reducing interfacial mobility, ultimately hindering nanoemulsion formation upon aqueous dilution [53]. Consequently, formulations exhibiting extensive gelation were excluded from further optimization studies. No external co-solvents or carrier solvents were used during excipient mixing. All excipients were directly mixed under gentle heating (40–45 °C) and magnetic stirring to facilitate homogenization and maintain a solvent-free lipid formulation system. The absence of additional organic solvents was intentional to minimize formulation complexity and improve translational applicability for oral delivery systems.

In this study, the term ‘undiluted’ refers to liquid SEDDS at lipid concentrations of 10–20% (*w*/*w*), which was selected to reflect physiologically relevant dispersion conditions following oral administration rather than analysis of the neat pre-concentrate. Preliminary experiments were conducted across a wider concentration range (1–50% *w*/*w*), and the 10–20% range was identified as optimal for maintaining representative self-emulsification behavior while remaining within the operational limits of the instrument [54,55].

To address potential issues of excessive scattering intensity and detector saturation, several measures were implemented. (1) Instrument configuration: Measurements were performed using backscatter detection (173°), which reduces multiple scattering effects and is well-suited for relatively concentrated dispersions. (2) Attenuation optimization: The instrument’s automatic attenuator adjusted the laser intensity to maintain photon count rates within the recommended range (typically 200–300 kcps), thereby preventing detector saturation [56]. (3) Reproducibility checks: Consistent droplet size and PDI values were obtained across replicate measurements and across the 10–20% (*w*/*w*) concentration range, indicating robustness of the measurements. (4) Viscosity and refractive index inputs: Appropriate dispersant parameters were used to ensure accurate data fitting under these conditions [57]. Importantly, dilution below this range was found to alter the microstructure of the system and, in some cases, led to changes in droplet size distribution, likely due to shifts in equilibrium during self-emulsification [58]. Therefore, analysis at 10–20% (*w*/*w*) was considered both analytically valid and more biorelevant.

Following optimization of the SEDDS excipient parameters, the resulting liquid and solid-dispersible (freeze-dried) SEDDS demonstrated relatively enhanced colloidal stability with finer, homogenous dispersions from the Labrafac PG-Capryol 90 formulations (Batch LC) of liquid and solid-dispersible formulations. The reduced droplet size of this batch of liquid and solid SEDDS portended advantages for subsequent manufacturing applications with respect to processing and encapsulation in capsules or compression into tablets, with the potential of improving the overall efficacy and stability of the oral drug delivery system. Phase behavior of the liquid and solid-dispersible SEDDS and their composite excipients through thermal analysis confirmed the formation of a stable amorphous system with potential for facilitating the entrapment and controlled-release of internalized therapeutic cargo. The thermal analysis (DSC) results highlighted the effect that the freeze-drying process had by reducing the crystalline nature and enhancing molecular dispersion of the excipient components within the system, with no evidence of significant incompatibility. The formation of an amorphous system is anticipated to improve the physicochemical properties, particularly solubility and dissolution behavior, of the developed formulation [18].

Sucrose was employed as a lyoprotectant to preserve the structural integrity of the double-emulsion system during freeze-drying and to facilitate the formation of a stable amorphous solid dispersion. Its protective effect is primarily attributed to the formation of a hydrogen-bonded glassy matrix, which stabilizes the interfacial film and prevents droplet coalescence and collapse during ice crystal formation and sublimation [59,60]. Upon reconstitution, this amorphous matrix readily dissolves, enabling rapid redispersion of the system and recovery of nano-sized droplets with minimal changes in droplet size and polydispersity. This behavior is consistent with the observed stability of the solid-dispersible SEDDS over extended storage periods.

In terms of biological performance, sucrose is generally considered inert and is not expected to directly interfere with cellular uptake mechanisms [61]. However, it may exert an indirect influence by modulating key physicochemical properties such as droplet size, surface characteristics, and dispersion kinetics, which are known to affect endocytic uptake pathways [62]. In the present study, the efficient intracellular delivery observed via fluorescence imaging suggests that the presence of sucrose did not adversely impact cellular internalization of either drug.

Despite its advantages, the use of sucrose as a cryoprotectant is associated with certain limitations. Its hygroscopic nature may lead to moisture uptake during storage, potentially affecting powder stability and redispersion behavior. Additionally, excessive concentrations can result in increased viscosity upon reconstitution or incomplete dispersion, which may negatively impact formulation performance [59]. To mitigate these challenges, careful optimization of cryoprotectant concentration was performed for the study, which resulted in a stable freeze-dried product.

A successful optimization and formation of a freeze-dried double-emulsion was achieved which serves proof-of-principle for single- or dual-drug delivery of solid dispersion of oral drug delivery, with the added advantage of extending the shelf life of the dosage form with easy access for the oral formulation [63].

Finally, while limited in its scope for modeling a more complex biological system for investigating the translational utility of the liquid or solid-dispersible SEDDS in a solid tumor, a MDA-MB-468 and MDA-MB-231 derived breast cancer spheroid were utilized as both a preliminary and endpoint model in this proof-of-principle study for assessing the ability of the SEDDS to facilitate internalization of doxorubicin and ellipticine as model therapeutic system. Importantly, fluorescence imaging with three layers of Z-stacking demonstrated deep penetration and co-localization of both doxorubicin and ellipticine within the breast cancer cells.

When considered together, the results provide mechanistic insight into how formulation variables govern system performance, as reflected in phase transitions, droplet characteristics, thermal behavior, and cellular uptake. The establishment of a structure–property–function relationship underscores the ability of the optimized SEDDS to achieve stable double-emulsion systems with improved encapsulation efficiency and delivery performance. These findings offer a rational basis for further optimization and support the translational potential of solid-dispersible SEDDS for oral administration. Future studies will extend this drug formulation and delivery concept to the design, biophysical and biomolecular characterization, and investigation of the drug efficacy of therapeutic-loaded solid dosage forms - capsules and compressed tablets - prepared using this SEDDS formulation strategy.

## 5. Conclusions

The present study successfully demonstrates a proof of principle for the development of a dual drug-loaded self-emulsifying drug delivery system (SEDDS) that can be transformed into a solid-dispersible dosage form via freeze-drying. Systematic excipient screening and the use of ternary phase diagrams enabled the identification of optimal compositional regions for stable w/o and w/o/w emulsions, highlighting the critical role of component ratios in formulation design. The optimized formulations, particularly those based on Labrafac PG—Capryol 90, exhibited improved colloidal stability, reduced droplet size, and homogeneous dispersion. Thermal analysis further confirmed the formation of a stable amorphous system, indicating improved molecular dispersion and the potential for enhanced solubility and controlled drug release. The incorporation of sucrose as a lyoprotectant proved effective in preserving emulsion integrity during freeze-drying and enabled efficient redispersion with minimal changes in physicochemical properties.

Importantly, the developed SEDDS demonstrated promising biological performance, as evidenced by efficient intracellular delivery and tumor-like spheroid penetration of the model drugs, doxorubicin and ellipticine. These findings suggest that the formulation strategy not only maintains physicochemical stability but also supports effective cellular uptake. Overall, this study establishes a robust and scalable platform for the development of solid-dispersible lipid-based formulations for oral delivery, with potential applications in single or combination drug therapy.

In summation, lipid-based drug delivery systems offer a powerful approach to overcoming the biopharmaceutical limitations of orally administered drugs, particularly those with poor aqueous solubility or low GI permeability. The integration of SEDDS technology with freeze-drying and w/o/w emulsion strategies provides a novel and effective platform for single- and dual-drug delivery, enabling simultaneous administration of hydrophilic and lipophilic compounds in a single, stable oral dosage form. This dual delivery system represents a promising advancement in the field of oral drug formulation, with the potential to significantly improve therapeutic outcomes, formulation stability, and patient adherence.

## Figures and Tables

**Table 1:** Screening for miscibility of oil excipients and dispersion characteristics in an aqueous phase.

Ratios		Aqueous Phase										
		10	9	8	7	6	5	4	3	2	1	0
**Oil Phase**	0	NA										
	1		Liquid									
	2			Liquid								
	3				Liquid							
	4					Liquid						
	5						Liquid					
	6							Gel				
	7								Gel			
	8									Gel		
	9										Gel	
	10											NA

Specific weight ratios of oil (Labrafac PG), surfactants (Labrasol and Gelucire 44/14), and co-surfactants (Span 80 or Capryol 90) were mixed to obtain four different homogenous oil phase mixtures (i.e., SEDDS 1–4) and each was dispersed with water at dilutions of 1:9 to 9:1 ratio of oil phase to aqueous phase, respectively. For example, for a SEDD1 oil phase mixture containing Labrafac PG, Labrasol, Gelucire 44/14, and Span 80, nine (9) dilutions in water were obtained corresponding to 1:9, 2:8, 3:7, 4:6, 5:5, 6:4, 7:3, 8:2, and 9:1.

**Table 2:** Optimization of oil–surfactant mix using Labrafac PG, Span 80, and Caproyl 90 for oil phase.

**Batch LS:** Labrafac PG and Span 80 Mix			
Oil Phase Mixture	Labrafac PG (mg) (Oil)	Span 80 (mg) (Surfactant)	Weight Ratio (Oil: Surfactant)
SEDDS 1	147.4	147.4	1:1
SEDDS 2	196.6	98.3	2:1
SEDDS 3	216.7	73.7	3:1
SEDDS 4	235.9	59.0	4:1
**Batch LC:** Labrafac PG and Caproyl 90 Mix			
Oil Phase Mixture	Labrafac PG (mg) (Oil)	Caproyl 90 (mg) (Surfactant)	Weight Ratio (Oil: Surfactant)
SEDDS 1	147.4	147.4	1:1
SEDDS 2	196.6	98.3	2:1
SEDDS 3	216.7	73.7	3:1
SEDDS 4	235.9	59.0	4:1

**Table 3:** Optimization of different ratios of primary (w/o) emulsions.

Formulations	Aqueous Phase			Oil Phase		
	Ratio	Parts	Weight (mg)	Ratio	Parts	Weight (mg)
A	1	100	100	9	900	942
B	2	200	200	8	800	838
C	3	300	300	7	700	733
D	4	400	400	6	600	628

**Table 4:** Composition of double emulsion groups.

Formulation No.	Oil Phase (mg)					Internal Aqueous (mg)	External Aqueous Phase (mg)	Total Weight (mg)	Total Oil Phase (% *w*/*w*)
	Labrafac PG	Caproyl 90	Gelucire 44/14	Labrasol	Total Oil Phase				
1.1.1	161.20	161.20	21.41	55.18	399	44. 33	3600	4043.33	9.87
1.2.1	161.20	161.20	21.41	55.18	399	99.75	3600	4098.75	9.73
2.1.1	214.93	107.46	21.41	55.18	399	44.33	3600	4043.33	9.87
2.2.1	214.93	107.46	21.41	55.18	399	99.75	3600	4098.75	9.74
1.1.2	322.40	322.40	42.83	110.36	798	88.67	3200	4086.67	19.53
1.2.2	429.87	214.93	42.83	110.36	798	199.5	3200	4197.5	19.01
2.1.2	322.40	322.40	42.83	110.36	798	88.67	3200	4086.67	19.53
2.2.2	429.87	214.93	42.83	110.36	798	199.5	3200	4197.5	19.53

**Table 5:** Characterization of w/o/w Double-Emulsion Containing Span 80 (Batch LS) and Caproyl 90 (Batch LC) at 0 days.

**Characterization of w/o/w Double-Emulsion Containing Span 80 (Batch LS)**						
	**Formulation No.**	**Oil Phase (mg)**	**Aqueous Phase (mg)**	**Zeta Potential**	**Droplet Size**	**PDI**
				**Mean ± SD (mV)**	**Mean ± SD (nm)**	**Mean**
Formulation A (1:9)	1.1.1	399	3600	−3.75 ± 1.24	2115 ± 0.51	0.005
	1.2.1	399	3600	−9.42 ± 5.62	2808 ± 1.24	0.005
	2.1.1	399	3600	−3.23 ± 1.01	1106 ± 0.31	0.005
	2.2.1	399	3600	−20.41 ± 1.85	804 ± 0.20	0.005
Formulation B (2:8)	1.1.2	798	3200	−49.56 ± 0.09	655 ± 0.08	0.005
	1.2.2	798	3200	−14.96 ± 1.34	1026 ± 0.16	0.295
	2.1.2	798	3200	−23.83 ± 5.71	883 ± 0.28	0.005
	2.2.2	798	3200	−20.81 ± 3.22	705 ± 2.06	0.005
**Characterization of w/o/w Double-Emulsion Containing Caproyl 90 (Batch LC)**						
	**Formulation No.**	**Oil Phase (mg)**	**Aqueous Phase (mg)**	**Zeta Potential**	**Droplet Size**	**PDI**
				**Mean ± SD (mV)**	**Mean ± SD (nm)**	**Mean**
Formulation A (1:9)	1.1.1	399	3600	−10.64 ± 0.67	89.65 ± 1.55	0.336
	1.2.1	399	3600	−2.61 ± 3.96	103.15 ± 11.05	0.299
	2.1.1	399	3600	1.74 ± 2.73	575.2 ± 46.1	0.005
	2.2.1	399	3600	2.38 ± 4.37	550.8 ± 207	0.012
Formulation B (2:8)	1.1.2	798	3200	−5.74 ± 3.88	571.5 ± 92.4	0.005
	1.2.2	798	3200	−5.94 ± 1.65	346.4 ± 19.7	0.005
	2.1.2	798	3200	−10.26 ± 0.44	329.85 ± 3.15	0.005
	2.2.2	798	3200	−7.00 ± 3.25	279.2 ± 6.3	0.242

**Table 6:** Characterization of w/o/w Double-Emulsion Containing Span 80 (Batch LS) and Caproyl 90 (Batch LC) after 28 days.

**Characterization of w/o/w Double-Emulsion Containing Span 80 (Batch LS) After 28 Days**					
	**Formulation No.**	**Oil Phase (mg)**	**Aqueous Phase (mg)**	**Droplet Size**	**PDI**
				**Mean ± SD (nm)**	**Mean**
Formulation A (1:9)	1.1.1	399	3600	2891.45 ± 30.57	0.234
	1.2.1	399	3600	3208.62 ± 25.89	0.786
	2.1.1	399	3600	5035.43 ± 54.36	0.921
	2.2.1	399	3600	7645.12 ± 46.27	0.954
Formulation B (2:8)	1.1.2	798	3200	2547.01 ± 24.80	0.123
	1.2.2	798	3200	2436.67 ± 49.66	0.214
	2.1.2	798	3200	4883.72 ± 15.28	0.763
	2.2.2	798	3200	5132.43 ± 21.35	0.876
**Characterization of w/o/w Double-Emulsion Containing Caproyl 90 (Batch LC) After 28 Days**					
	**Formulation No.**	**Oil Phase (mg)**	**Aqueous Phase (mg)**	**Droplet Size**	**PDI**
				**Mean ± SD (nm)**	**Mean**
Formulation A (1:9)	1.1.1	399	3600	213.12 ± 13.51	0.245
	1.2.1	399	3600	345.12 ± 19.24	0.114
	2.1.1	399	3600	565.02 ± 23.10	0.356
	2.2.1	399	3600	665.21 ± 18.72	0.154
Formulation B (2:8)	1.1.2	798	3200	612.76 ± 16.40	0.015
	1.2.2	798	3200	412.40 ± 20.71	0.026
	2.1.2	798	3200	554.34 ± 14.15	0.389
	2.2.2	798	3200	456.49 ± 45.3	0.576

## Data Availability

The original contributions presented in this study are included in the article. Further inquiries can be directed to the corresponding author.

## Abbreviations

The following abbreviations are used in this manuscript:

APIs	Active pharmaceutical ingredients
DAPI	4′,6-diamidino-2-phenylindole
DSC	Differential scanning calorimetry
FDA	Food and Drug Administration
GI	Gastrointestinal
HLB	Hydrophile–lipophile balance
LBDDS	Lipid-based drug delivery systems
NLCs	Nanostructured lipid carriers
o/w	Oil-in-water
PDI	Polydispersity index
PSN	Penicillin, streptomycin, and neomycin
RHLB	Required hydrophile–lipophile balance
SEDDS	Self-emulsifying drug delivery system
SLNs	Solid lipid nanoparticles
SMEDDS	Self-micro-emulsifying drug delivery system
SNEDDS	Self-nano-emulsifying drug delivery system
w/o	Water-in-oil
w/o/w	Water-in-oil-in-water
